# Nutrients: Signal 4 in T cell immunity

**DOI:** 10.1084/jem.20221839

**Published:** 2024-02-27

**Authors:** Jana L. Raynor, Hongbo Chi

**Affiliations:** 1 Department of Immunology, St. Jude Children’s Research Hospital, Memphis, TN, USA

## Abstract

T cells are integral in mediating adaptive immunity to infection, autoimmunity, and cancer. Upon immune challenge, T cells exit from a quiescent state, followed by clonal expansion and effector differentiation. These processes are shaped by three established immune signals, namely antigen stimulation (Signal 1), costimulation (Signal 2), and cytokines (Signal 3). Emerging findings reveal that nutrients, including glucose, amino acids, and lipids, are crucial regulators of T cell responses and interplay with Signals 1–3, highlighting nutrients as Signal 4 to license T cell immunity. Here, we first summarize the functional importance of Signal 4 and the underlying mechanisms of nutrient transport, sensing, and signaling in orchestrating T cell activation and quiescence exit. We also discuss the roles of nutrients in programming T cell differentiation and functional fitness and how nutrients can be targeted to improve disease therapy. Understanding how T cells respond to Signal 4 nutrients in microenvironments will provide insights into context-dependent functions of adaptive immunity and therapeutic interventions.

## Teaser

Nutrients interplay with Signal 1 (antigens), Signal 2 (co-stimulation), and Signal 3 (cytokines) to orchestrate T cell activation, differentiation, and function. This review highlights the signaling and functional effects of nutrients as Signal 4 in T cell–mediated immunity.

## Introduction

T cell immunity is integral to host health by providing protective immunity to infection and cancer and maintaining tissue homeostasis. T cells develop in the thymus, and upon maturation, enter secondary lymphoid tissues as quiescent naïve T cells with low metabolic, transcriptional, and translational activities (Chapman et al., 2020). During adaptive immune responses, naïve T cells exit quiescence and become activated to undergo clonal expansion and lineage differentiation, a process initiated by three well-established signaling events: T cell receptor (TCR) binding to antigen presented on major histocompatibility complex (MHC) molecules (Signal 1), costimulation largely mediated by CD28 (Signal 2), and certain cytokine signals (Signal 3). Advances in our understanding of how nutrients, including glucose, amino acids, and lipids, orchestrate T cell responses have revealed key roles for these factors in T cell activation, differentiation, and function, in coordination with Signals 1–3, thereby collectively establishing nutrients as Signal 4 in adaptive immunity.

Beyond shaping cellular activation, nutrients impact T cell fate decisions and functional specialization. For instance, upon activation, naïve CD4^+^ T cells differentiate into distinct T helper (Th) subsets, including Th1, Th2, Th17, and regulatory T (Treg) cells, a process influenced by nutrients and microenvironmental cues that fuel and support their unique metabolic requirements. Further, nutrients help orchestrate effector CD4^+^ and CD8^+^ T cell responses and memory formation in different disease contexts. Although naïve T cell activation occurs in lymphoid tissues that are considered to be replete with nutrients essential for T cell activation, different T cell populations undergo functional specialization that allows them to combat infectious agents or tumors in discrete tissue sites, and therefore cells must adapt to nutrient and environmental signals for effective responses. Under conditions of cellular activation, differentiation, or tissue adaptation, T cells integrate nutrients as Signal 4 through a three-tiered process composed of nutrient transport, sensing, and signaling (Long et al., 2021), thus allowing the cells to sense and respond to changes in nutrient availability. Harnessing nutrient-mediated regulation of T cell immunity in tissue and disease-specific contexts is emerging as a new strategy for disease therapy (Collins and Belkaid, 2022; Giles et al., 2023; Kao et al., 2022).

In this review, we summarize the impacts of nutrients and associated factors (e.g., nutrient-derived metabolic intermediates and extracellular metabolites) on T cell activation, differentiation, and function, and the underlying mechanisms. First, we describe the contributions of nutrients as Signal 4 to T cell activation and quiescence exit. Next, we detail how nutrients shape T cell fate (e.g., T cell effector subset differentiation and memory cell formation) and function (e.g., antitumor and pathogen responses). We then discuss how nutrient deprivation and inhibitory nutrients within microenvironments limit T cell function. Finally, we review how targeting nutrients can be harnessed for disease therapy and conclude by discussing future directions and implications for nutrients as Signal 4 in licensing T cell immunity.

## Nutrients license T cell activation

Nutrients regulate T cell activation, in part by interplaying with Signals 1–3 to drive T cell immunity (Chapman and Chi, 2022; Giles et al., 2023; Long et al., 2021; Shi et al., 2019). The mechanistic basis for the three-tiered process by which nutrients serve as Signal 4 for T cell activation, including the molecular processes regulating nutrient uptake or transport, sensing, and signal transduction (Long et al., 2021), is emerging and discussed below.

### Nutrient transport

**Figure 1.**
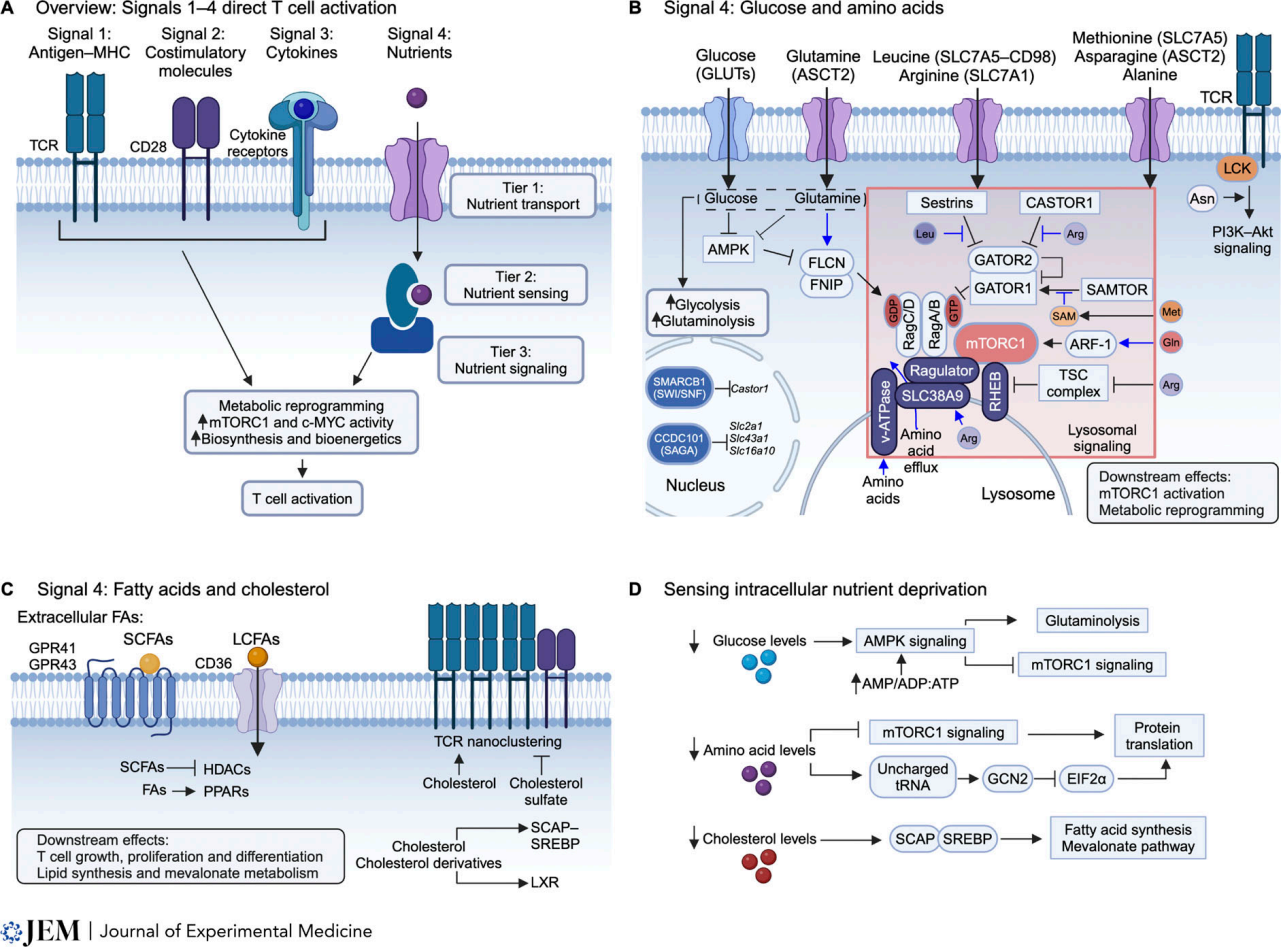
**Signal 4 nutrients in directing T cell activation. (A)** Overview of Signals 1 (TCR binding to antigen presented on MHC molecules), 2 (co-stimulation by CD28), 3 (cytokine signals), and 4 (nutrients) that drive T cell immunity. Signal 4 is mediated through a three-tiered process composed of nutrient transport, sensing, and signal transduction. Signals 1–3 can augment Signal 4 by promoting the expression of nutrient transporters, while Signal 4 also interplays with Signals 1–3, for example, by shaping signaling and metabolic events. Integration of Signals 1–4 results in metabolic reprogramming, associated with increased mTORC1 signaling, c-MYC activity, and activation of biosynthesis pathways and cellular bioenergetics, altogether driving T cell activation. **(B)** Glucose and amino acid uptake into T cells, mediated by membrane transporters, promotes mTORC1 activation and metabolic reprogramming. Sestrins, CASTOR1, and SAMTOR represent cytosolic sensors of amino acids. Leucine and arginine respectively bind to and sequester Sestrins and CASTOR1 from GATOR2, relieving their suppressive effects on GATOR2, thereby allowing GATOR2 to promote mTORC1 activation (via inhibiting GATOR1). SAMTOR senses the methionine metabolite S-adenosylmethionine (SAM). SAM binding to SAMTOR disrupts SAMTOR–GATOR1 complex formation, thereby inhibiting the ability of GATOR1 to negatively regulate mTORC1 activation. SLC38A9 senses arginine in the lysosome, and both SLC38A9 and v-ATPase signal the increase in intralysosomal amino acid concentrations to promote mTORC1 activation, which may involve controlling the efflux of amino acids from the lysosome. Arginine also promotes mTORC1 signaling by regulating TSC–RHEB signaling. Glutamine signals through ADP ribosylation factor 1 (ARF-1) to promote mTORC1. Asparagine is sensed by LCK to promote TCR-mediated PI3K–Akt signaling. The SWI/SNF complex (including SMARCB1) inhibits gene expression of *Castor1* and thereby enhances mTORC1 activity. CCDC101-associated SAGA complex inhibits the expression of glucose and amino acid transporters genes (*Slc2a1*, *Slc43a1*, and *Slc16a10*) and maintains T cell quiescence. Positive regulators of mTORC1 are denoted in ovals and negative regulators of mTORC1 are denoted in rectangles. Blue arrows indicate nutrient sensing that remains to be validated in primary T cells. **(C)** Fatty acid (FA) and cholesterol sensing and signaling can promote T cell growth, proliferation, and differentiation. SCFAs signal through GPCRs, or intracellular SCFAs can act as HDAC inhibitors. LCFAs can be transported into cells by CD36 and sensed intracellularly by PPARs. Cholesterol and cholesterol sulfate regulate TCR nanoclustering to either promote or impair TCR signaling, respectively. Intracellular cholesterol and cholesterol derivatives are recognized and can signal through SCAP–SREBP to influence lipid synthesis and mevalonate metabolism. Cholesterol derivatives are recognized and can signal through LXR to regulate T cell differentiation. **(D)** Mechanisms to sense low intracellular nutrient and metabolite abundance are also present in T cells. Glucose or glutamine deprivation activates AMPK in T cells, and AMPK mediates increased glutaminolysis and reduced mTORC1 signaling during glucose deprivation. AMPK is activated when the levels of AMP or ADP are relatively higher than ATP, or by extracellular ATP indirectly (not depicted here). Low amino acid levels impair mTORC1 signaling and increase the number of uncharged tRNAs. General control nonderepressible 2 (GCN2) binds to uncharged tRNAs and inhibits eukaryotic translation initiator factor 2 α (EIF2α)–dependent protein translation. Low cholesterol levels activate SCAP–SREBP signaling, which promotes fatty acid synthesis and the mevalonate pathway by transcriptional induction of lipid biosynthetic enzyme expression.

T cell activation requires the uptake of certain extracellular nutrients and coordination of intracellular metabolism, which enables cells to meet the bioenergetic and biosynthetic requirements for cell growth and proliferation (Fig. 1 A). The uptake and metabolism of glucose are required for T cell activation (Jacobs et al., 2008; Wang et al., 2011), which are coordinately regulated (Chapman et al., 2020). The increased demands for nutrients are met in part by upregulated transporter expression, which requires Signals 1 and 2. For instance, glucose transporter 1 (GLUT1) expression is upregulated by the combined actions of TCR and CD28 signaling, leading to enhanced glucose uptake (Frauwirth et al., 2002; Jacobs et al., 2008). Further, Raptor-mediated mTORC1 signaling integrates TCR–CD28 signals with metabolic reprogramming, including increased glucose metabolism, to promote T cell quiescence exit and proliferation (Yang et al., 2013). Unlike activated T cells, quiescent T cells do not require GLUT1 expression for cell survival, although GLUT2, GLUT3, and GLUT6 may play redundant roles (Fu et al., 2023; Macintyre et al., 2014). GLUT2 expression also contributes to optimal CD8^+^ T cell responses, and its expression is regulated by environmental factors, including glucose levels (Fu et al., 2023). Altogether, signal-dependent regulation of glucose transporter expression may coordinate the rate of glucose uptake with the maintenance of quiescence versus quiescence exit and cell activation, and the initiation of quiescence exit relies in part on the coordinated regulation of glucose transporter expression by Signals 1 and 2.

In addition to glucose, amino acid uptake facilitates T cell activation, in large part by activating mTORC1 (Chapman et al., 2020; Huang et al., 2020). TCR signals promote the expression of many system L amino acid transporters, including the heterodimer transporter SLC7A5–CD98 that facilitates the import of large neutral amino acids into cells, with SLC7A5 deficiency impairing mTORC1 signaling and T cell activation (Howden et al., 2019; Sinclair et al., 2013). Further, transporters of glutamine and leucine, including ASCT2 (encoded by *Slc1a5*) and SNAT1 (encoded by *Slc38a1*), couple TCR–CD28 signaling with glutamine and leucine uptake (Carr et al., 2010; Nakaya et al., 2014). Uptake of additional amino acids, including methionine (via SLC7A5), asparagine (via ASCT2), and alanine (likely through SNAT1), also promote T cell activation and cytokine production (Ron-Harel et al., 2019; Sinclair et al., 2019; Wu et al., 2021). Conversely, limiting the upregulation of glucose and amino acid transporters through the CCDC101-associated SAGA complex restrains TCR–CD28 and glucose-induced mTORC1 signaling to maintain T cell quiescence (Long et al., 2021). In addition to Signals 1 and 2, Signal 3 (e.g., IL-7, IL-2, and IL-33) promotes glucose uptake and amino acid transporter expression, supporting glycolytic flux in T cells (Jacobs et al., 2010; Liang et al., 2022; Pearson et al., 2012; Rathmell et al., 2001; Wofford et al., 2008). For instance, IL-7 promotes glucose metabolism to promote resting T cell survival (Jacobs et al., 2010; Rathmell et al., 2001; Wofford et al., 2008). Also, once T cells are activated, IL-2 signals help to sustain the expression of GLUT1, GLUT3, and system L amino acid transporters (Rollings et al., 2018; Sinclair et al., 2013). Importantly, the roles of glucose and amino acids in T cell activation are coordinated, as T cells require amino acid transporter expression for optimal GLUT1 expression, glucose uptake, and upregulation of glycolysis (Nakaya et al., 2014; Sinclair et al., 2013), likely through sustaining TCR-induced mTORC1 and c-MYC activity (Nakaya et al., 2014; Shi et al., 2019; Sinclair et al., 2019). Interestingly, transgenic expression of GLUT1 is sufficient to alter Treg cell accumulation and function in vivo (Gerriets et al., 2016). Further, increasing nutrient signaling or transporter expression correlates with altered T cell homeostasis (Long et al., 2021; Yang et al., 2011). Collectively, these studies establish a role for Signal 4 in modulating T cell activation and homeostasis.

Furthermore, fatty acid uptake and biosynthesis contribute to T cell activation, proliferation, and survival, as these processes are impaired in CD4^+^ T cells cultured in fatty acid–free conditions or in the presence of a pharmacological inhibitor of ACC1 (5-[tetradecyloxy]-2-furoic acid) and are restored with the supplementation of extrinsic fatty acids. Mechanistically, activation of transcription factor PPAR-γ by TCR–CD28–mTORC1 signaling induces the expression of fatty acid transporters, and pharmacological inhibition or genetic deletion of PPAR-γ impairs fatty acid uptake and cell proliferation (Angela et al., 2016). Finally, the import of minerals, including magnesium, potassium, iron, and calcium, impact T cell quiescence exit and activation (Chapman et al., 2020). Collectively, nutrient uptake is coordinated with Signals 1–3 and contributes to T cell exit from quiescence and productive activation.

### Nutrient sensing and signaling

To detect the intracellular levels of amino acids, sugars, or lipids, cells express proteins that bind to nutrients (or their downstream metabolites) and induce downstream signaling events, a process referred to as sensing. Amino acid sensing predominately occurs at the lysosome to regulate mTORC1 signaling (Fig. 1 B), a master controller of T cell quiescence exit (Chapman et al., 2020; Tan et al., 2017; Yang et al., 2013). Mechanistically, amino acid abundance within the lysosome regulates mTORC1 signaling through an inside-out signaling mechanism mediated by vacuolar H^+^-ATPase and SLC38A9 (Abu-Remaileh et al., 2017; Jung et al., 2015; Rebsamen et al., 2015; Wang et al., 2015b; Wyant et al., 2017; Zoncu et al., 2011). Further, cytosolic amino acid sensors for leucine, arginine, and methionine (namely, its downstream metabolite S-adenosylmethionine [SAM]) have been identified, including Sestrins, CASTOR1, and SAMTOR, respectively (Chantranupong et al., 2016; Gu et al., 2017; Peng et al., 2014; Saxton et al., 2016a, 2016b; Wolfson et al., 2016). Sestrins, CASTOR1, and SAMTOR are upstream regulators of GATOR1 and GATOR2 complexes. GATOR1 negatively regulates mTORC1 activation by functioning as a GTPase-activating protein (GAP) for RagA/B and promoting the GDP-bound form of RagA or Rag B (RagA/B), thereby inhibiting the Rag GTPase complex (Bar-Peled et al., 2013). In contrast, GATOR2 binding to GATOR1 impairs the GAP activity of GATOR1 and acts as a positive regulator of mTORC1 (Bar-Peled et al., 2013). Leucine and arginine binding to Sestrins or CASTOR1, respectively, disrupts their interaction with GATOR2, and consequently relieves the suppressive effects and enables GATOR2 (via inhibiting GATOR1) to promote mTORC1 activation (Chantranupong et al., 2016; Saxton et al., 2016a, 2016b; Wolfson et al., 2016). SAM binding to SAMTOR disrupts the interaction with GATOR1 and KICSTOR, leading to mTORC1 activation (Gu et al., 2017). Finally, glutamine promotes mTORC1 activation through an alternative GTPase, ARF-1 (Jewell et al., 2015). Although the functional importance of the majority of these sensors remains largely unresolved in T cells, we recently found that the SWI/SNF complex promotes mTORC1 signaling by repressing TCR–CD28-induced expression of CASTOR1 (Long et al., 2021). Further, Sestrins contribute to impaired T cell function that is observed during aging (Lanna et al., 2017; Pereira et al., 2020), and the involvement of nutrients in this process remains to be ascertained. How nutrient sensing by these lysosome-associated sensors and signaling complexes contributes to T cell activation and function requires further investigation.

Several important signal transducers mediate downstream events of these amino acid sensors to promote mTORC1 signaling. Rag GTPase complex, composed of RagA/B paired with RagC or RagD (RagC/D), promotes the recruitment and activation of mTORC1 at the lysosome when amino acid levels are sufficient (Kim et al., 2008; Sancak et al., 2008, 2010). RagA also senses intracellular glucose abundance to affect mTORC1 recruitment and activation at the lysosome (Efeyan et al., 2013). In addition, glutaminolysis (likely via α-ketoglutarate [α-KG] production) promotes GTP loading of RagB, leading to mTORC1 localization to the lysosome and subsequent activation (Durán et al., 2012). T cells require the interaction between SEC31A and SEC13, a component of GATOR2, to function upstream of GATOR1 and RagA for amino acid and glucose-mediated mTORC1 activation (Long et al., 2021). Further, RagA/B proteins are necessary for amino acid signaling to license and sustain TCR-induced mTORC1 activity for Treg cell activation, proliferation, and functional programming (Do et al., 2020; Shi et al., 2019). Arginine promotes mTORC1 signaling in Treg cells by disrupting tuberous sclerosis 2 (TSC2, a component of the TSC complex) association with the lysosome (Shi et al., 2019), which is likely permissive for RHEB-dependent mTORC1 activation (Demetriades et al., 2014). These events are at least partially dependent on RagA/B, although PI3K–Akt-dependent phosphorylation of TSC2, such as that induced by TCR–CD28 stimulation, also contributes to these effects (Menon et al., 2014; Shi et al., 2019; Yang et al., 2011). Further, removal of amino acids or glucose from activated T cell populations dampens mTORC1 signaling but their add-back induces robust mTORC1 activation (Long et al., 2021; Shi et al., 2019), supporting that these nutrients help maintain mTORC1 signaling in activated T cells. Thus, there is an extensive interplay between Signal 4 (especially mediated by amino acids) and Signals 1 and 2 in T cells.

Additional molecular mediators integrate nutrient signals to promote T cell signaling and functions. The SRC-family protein tyrosine kinase LCK senses intracellular asparagine levels, conferring enhanced LCK activity and TCR signaling to promote T cell activation and effector function (Wu et al., 2021). Further, the uptake of glutamine downstream of TCR–CD28 signaling requires the CBM complex, composed of CARMA1, BCL10, and MALT1, which promotes ASCT2-mediated glutamine uptake (Nakaya et al., 2014). Additionally, three transcriptional regulators, BAZ1B, PSIP1, and TSN, sense intracellular arginine and promote T cell survival (Geiger et al., 2016). Also, glutaminase (GLS), which converts glutamine to glutamate, promotes PIK3IP1 expression, thereby reducing IL-2–mediated mTORC1 signaling in Th1 cells (Johnson et al., 2018). In type-1 conventional dendritic cells (cDC1s), glutamine modulates cellular function through the folliculin (FLCN)–FNIP complex, which impedes activation of the nutrient-stress-responsive transcription factor, TFEB (Guo et al., 2023). Of note, the FLCN–FNIP complex is an upstream regulator of the Rag GTPase complex, promoting Rag GTPase-mediated mTORC1 activation when amino acid levels are sufficient (Kim and Guan, 2019; Petit et al., 2013; Tsun et al., 2013), and the contribution of FLCN–FNIP to sensing nutrient levels and downstream signaling remains to be explored in T cells.

Extracellular fatty acids bind and signal through G protein–coupled receptors (GPCRs), including GPR43 and GPR41, to regulate T cell responses (Fig. 1 C) (Maslowski et al., 2009; Smith et al., 2013; Sun et al., 2018; Trompette et al., 2018). Further, intracellular fatty acids are recognized by peroxisome proliferator-activated receptors (PPARs), which promote T cell activation, proliferation, and survival (Angela et al., 2016; Housley et al., 2011). Cholesterol is sensed by SLC38A9 or lysosomal GPCR-like protein (LYCHOS) at the lysosomal surface, enabling mTORC1 activation (Castellano et al., 2017; Shin et al., 2022), but the roles of these sensors in T cell responses require investigation. Further, intracellular cholesterol levels are sensed by SREBP cleavage activation protein (SCAP). Low levels of cholesterol activate SCAP to promote SREBP transcriptional activity. SREBPs mediate transcriptional induction of metabolic enzymes in de novo fatty acid and cholesterol synthesis and promote CD8^+^ T cell growth and antiviral responses, as well as intratumoral Treg cell function (Kidani et al., 2013; Lim et al., 2021). The addition of cholesterol overcomes the cell size and proliferation defects of SCAP-deficient CD8^+^ T cells, indicating that de novo lipid synthesis contributes to cell growth and proliferation during quiescence exit (Kidani et al., 2013). Intracellular cholesterol derivatives (e.g., oxysterols) are sensed by the liver X receptor (LXR) transcription factor, which influences T cell proliferation and CD4^+^ T cell subset differentiation (Bensinger et al., 2008; Lim et al., 2022). Together, these studies establish lipid sensing and downstream signaling pathways as important regulators of T cell activation and proliferation.

Cholesterol, along with the lipid sphingomyelin, in the plasma membrane serves as an important positive regulator of TCR signaling (Signal 1) by aiding TCR clustering and the formation of the immunological synapse (Molnár et al., 2012; Yang et al., 2016). Increasing plasma membrane cholesterol levels in CD8^+^ T cells by ablating ACAT1, a cholesterol esterification enzyme, enhances TCR signaling and promotes antitumor immunity (Yang et al., 2016). In contrast, cholesterol sulfate (CS), a cholesterol analog, disrupts cholesterol–TCR interactions to limit TCR signaling and T cell activation. During thymocyte development, CS levels are lowest in double-positive (DP) thymocytes. Further, DP thymocytes are more sensitive to CS-mediated inhibition of TCR signaling and promotion of cell death, likely via inhibiting positive selection (Wang et al., 2016). Cholesterol also functions to limit TCR signaling by binding to the inactive conformation of TCRβ, and disruption of cholesterol–TCRβ binding promotes the active conformation of TCR that is primed for signaling upon binding to peptide–MHC (Swamy et al., 2016). Structural analysis revealed that cholesterol and CS “latch” CD3ζ in an inactive conformation in the membrane, thereby inhibiting TCR–CD3-mediated signaling (Chen et al., 2022). Thus, cholesterol modulates TCR-mediated signaling events during initial T cell activation. Beyond cholesterol, membrane phospholipids of the phosphoinositide (PIP_n_) family regulate TCR signaling at different stages of the T cell response. Polyunsaturated PIP_2_ mediates TCR signaling after early T cell activation, while de novo synthesis of saturated PIP_2_, which requires glucose as a substrate, helps sustain signaling downstream of TCR to support effector T cell function (Edwards-Hicks et al., 2023). During thymocyte development, PIP_2_ replenishment by Nir3 is required for T cell maturation by promoting calcium signaling in response to TCR (Lu et al., 2023). Altogether, cholesterol and membrane lipids tune TCR signaling, providing an important checkpoint during selection in the thymus, and can balance T cell quiescence versus activation in the context of infection, cancer, and autoimmunity.

T cells also employ mechanisms to sense and adapt to low abundance of intracellular nutrients (Fig. 1 D) and altered extracellular metabolites. For instance, low intracellular amino acid levels increase the number of uncharged transfer ribonucleic acids (tRNAs). General control nonderepressible 2 (GCN2) binds to these uncharged tRNAs, leading to the inhibition of eukaryotic translation initiator factor 2 α (EIF2α)–dependent protein translation. Elevated GCN2 kinase activity arrests T cell proliferation (Chapman et al., 2020; Munn et al., 2005). In contrast, deletion of GCN2 expression allows T cells to escape suppression by indoleamine 2,3-dioxygenase (IDO)–expressing DCs (Munn et al., 2005), suggesting that sensing of low amino acid availability (including tryptophan) by GCN2 may serve as a mechanism for immune tolerance. Further, AMP-activated protein kinase (AMPK) is activated when the levels of AMP and ADP are high, relative to ATP. In T cells, glucose or glutamine deprivation also activates AMPK (Blagih et al., 2015). In turn, under glucose starvation conditions, AMPKα1 boosts glutamine metabolism in supporting T cell bioenergetics and survival (Rolf et al., 2013; Blagih et al., 2015). Also, extracellular ATP signals through the purinergic receptor P2RX7, leading to the activation of AMPK (Borges da Silva et al., 2018). AMPK signaling limits mTORC1 signaling and promotes the formation of metabolically quiescent memory T cells (Pearce et al., 2009; Rolf et al., 2013; Borges da Silva et al., 2018) and also is essential for sustaining effector T cell responses to viral and bacterial infections (Blagih et al., 2015). Therefore, mechanisms that sense lower nutrient abundance likely allow for metabolic flexibility to maintain T cell function under conditions of nutrient limitation.

## Nutrients shape T cell differentiation and function

In addition to naïve T cell activation, nutrients interplay with Signals 1–3 to control the function, subset specification, and longevity of antigen-experienced T cells. Specifically, CD4^+^ T cell differentiation into distinct subsets is regulated by microenvironmental cues (Saravia et al., 2019), including cytokines (Signal 3) and nutrients (Signal 4). Further, nutrients help orchestrate effector CD4^+^ and CD8^+^ T cell responses and memory formation in different disease contexts. Here, we summarize how glucose, amino acids, fatty acids, and cholesterol regulate T cell fate and promote functional responses, and the interplay of nutrients with cytokine signaling.

### Glucose

**Figure 2.**
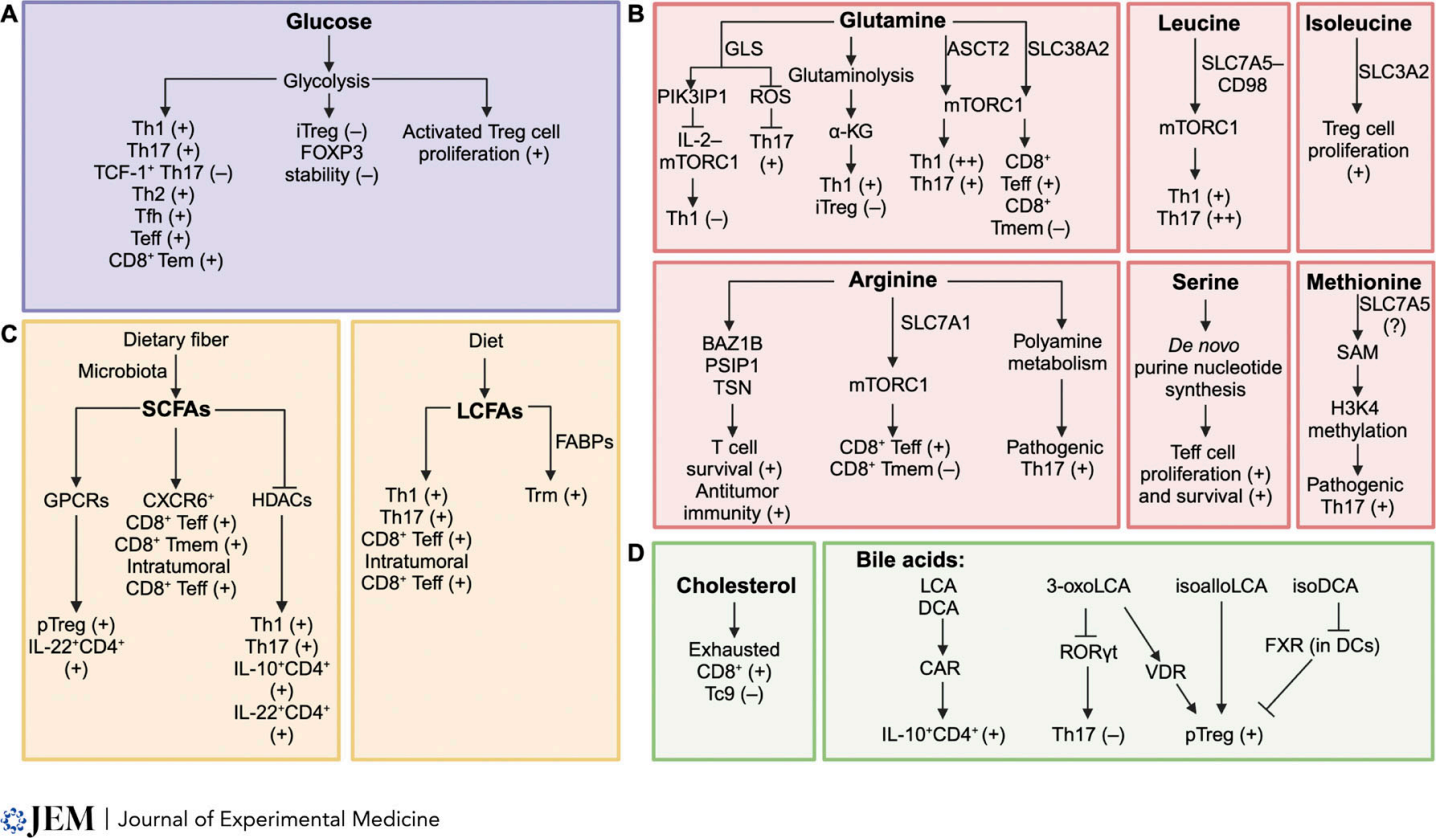
**Nutrients modulate T cell differentiation and function. (A)** Glucose metabolism (via glycolysis) promotes the differentiation of the indicated CD4^+^ T cell subsets and the generation of CD4^+^ and CD8^+^ effector T cells (Teff) and Tem CD8^+^ T cells. Glycolysis limits FOXP3 expression or stability but is upregulated in activated, proliferating Treg cells. **(B)** The contributions of intracellular amino acids (glutamine, leucine, isoleucine, arginine, serine, and methionine) to CD4^+^ T cell differentiation and Teff and memory (Tmem) CD8^+^ T cell responses are indicated. **(C)** SCFAs, including acetate, butyrate, and propionate, are derived from the fermentation of dietary fiber by intestinal microbiota. SCFAs alter the differentiation of the indicated CD4^+^ and CD8^+^ T cell subsets, which can occur via signaling through GPCRs or inhibiting HDACs. Diet-derived LCFAs regulate the indicated T cell populations. LCFAs promote Trm cell formation through fatty acid binding proteins (FABPs). Further, dietary TVA and linoleic acid, which are both LCFAs, enhance CD8^+^ T cell function and antitumor immunity. **(D)** Cholesterol promotes CD8^+^ T cell exhaustion and inhibits Tc9 cell generation in the TME. Intestinal bile acid derivatives (3-oxoLCA, isoalloLCA, and isoDCA) exert differential effects on the generation of Th17 and pTreg cells, as indicated. In particular, bile acid metabolites, including 3-oxoLCA, can promote pTreg cell accumulation in the colon through the vitamin D receptor (VDR). Constitutive androstane receptor (CAR) limits inflammation by detoxifying bile acids and promoting IL-10–producing CD4^+^ T cells in the intestine. Farnesoid X receptor (FXR) in DCs is suppressed by isoDCA, thereby promoting pTreg cell differentiation.

Beyond T cell activation, glucose uptake is also important for the differentiation of effector CD4^+^ T cell subsets (Fig. 2 A). Th1, Th2, and Th17 cells are highly glycolytic compared with in vitro inducible Treg (iTreg) cells, and glycolysis is essential to promote Th1 and Th17 cell generation (Gerriets et al., 2015; Michalek et al., 2011; Shi et al., 2011). Accordingly, deletion of HIF-1α, a key transcription factor mediating glycolytic enzyme expression, impairs Th17 cell generation (e.g., as evidenced by reduced IL-17 and IL-23R expression) and function in driving disease pathogenesis in experimental autoimmune encephalomyelitis (EAE) (Shi et al., 2011). Also in EAE, inhibition of glycolysis or mTORC1 activity via Raptor deletion promotes the generation of a TCF-1^+^ Th17 population with stemness-associated features that fail to induce autoimmunity (Karmaus et al., 2019). Further, Th1 and Th17 differentiation require GLUT1 expression (Macintyre et al., 2014), and GLUT3 contributes to Th17 cell effector function (Hochrein et al., 2022). Tfh cells also express more GLUT1 compared with non-Tfh cells, and GLUT1 overexpression promotes the generation of Tfh cells (Zeng et al., 2016). Importantly, the regulation of mTORC1 signaling and GLUT1 expression is bidirectional, as glucose uptake promotes mTORC1 activity during T cell activation, and mTORC1 activity contributes to GLUT1 upregulation in activated T cells (Zeng et al., 2016). Altogether, these studies show that activated T cell subsets have different dependencies on glucose metabolism.

Glucose and glucose metabolism play a pivotal role in directing Treg cells. Inhibiting the glycolytic pathway using 2-deoxyglucose (2-DG) promotes iTreg cell generation (Shi et al., 2011). Further, iTreg cells express lower levels of GLUT1 compared with effector T cells (Macintyre et al., 2014; Michalek et al., 2011). Reduced GLUT1 expression on iTreg cells is likely due to elevated AMPK activity (Macintyre et al., 2014; Michalek et al., 2011), as well as FOXP3 activity in human Treg cells, which limits GLUT1 expression by inhibiting Akt activation (Basu et al., 2015). Although GLUT1 deficiency does not affect iTreg cell suppressive function (Macintyre et al., 2014), GLUT1 overexpression reduces FOXP3 expression and the suppressive ability of iTreg cells and in vivo thymic-derived Treg (tTreg) cells (Gerriets et al., 2016), indicative of reduced Treg cell lineage stability. These findings are consistent with other studies that aberrant glycolysis is associated with impaired Treg cell function and lineage stability (Huynh et al., 2015; Shrestha et al., 2015; Wei et al., 2016). In contrast, glycolysis, through the glycolytic enzyme ENOLASE 1, induces expression of the *FOXP3* splice variant containing exon 2 to promote human iTreg cell differentiation and suppressive function (De Rosa et al., 2015). Further, proliferating tTreg cells show elevated GLUT1 expression, glucose uptake, and glycolysis compared with non-proliferating mouse and human tTreg cells (Gerriets et al., 2016; Procaccini et al., 2016). These studies reveal that while glucose and glycolysis have inhibitory effects on Treg cell generation and suppressive functions, they still contribute to Treg cell expansion. Thus, relative to other T cell subsets, the levels of glycolytic metabolism and upstream mTORC1 signaling must be tightly regulated to maintain Treg cell function (Chapman et al., 2020; Zeng and Chi, 2017).

Although memory T cells can fuel mitochondrial metabolism via fatty acid oxidation (FAO), glucose and glycolysis also contribute to memory T cell responses (Chapman and Chi, 2022). Indeed, NOTCH signaling increases GLUT1 expression to aid in glucose uptake and metabolism for memory CD4^+^ T cell survival (Maekawa et al., 2015). Human memory T cells also express GLUT1, and cells expressing higher levels of GLUT1 show stronger effector memory (Tem) T cell phenotypes (Cretenet et al., 2016). Enforced activation of HIF-1α via VHL deletion in CD8^+^ T cells enhances glycolysis to maintain ATP levels and boost CD8^+^ Tem cell formation, despite reduced mitochondrial oxidative phosphorylation (OXPHOS) (Phan et al., 2016). Further, compared with effector CD8^+^ T cells, in vitro IL-15–induced memory-like CD8^+^ T cells take up fewer extracellular fatty acids and therefore rely more on glucose for de novo fatty acid synthesis to fuel mitochondrial FAO (O’Sullivan et al., 2014). Also, GDP–fucose bioavailability is dependent upon glucose and fucose metabolism and further contributes to downstream NOTCH–Rbpj signaling, and perturbation of this nutrient signaling axis blocks CD8^+^ T cell terminal effector differentiation but promotes memory cell generation (Huang et al., 2021). Interestingly, under conditions of physiological carbon sources (i.e., medium with metabolite concentrations similar to mouse serum), CD8^+^ T cells consume less glucose and utilize other carbon sources, including lactate, to fuel mitochondrial metabolism (Kaymak et al., 2022), suggesting that T cells adapt to changes in extracellular nutrient availability. Together, these findings suggest that T cells alter nutrient usage and metabolic programs to support memory development.

Signal 3 also affects nutrient transport and downstream signaling. For instance, the H9T IL-2 “superkine” and IL-15 dampen the expression of glucose transporters and glucose uptake, resulting in reduced glycolysis, increased mitochondrial fitness, and enhanced CD8^+^ T cell stemness that improves antitumor activity (Alizadeh et al., 2019; Mo et al., 2021). Further, CD8^+^ T cell treatment with an IL-10-Fc fusion protein enhances pyruvate-dependent OXPHOS via the mitochondrial pyruvate carrier and reinvigorates antitumor responses (Guo et al., 2021). IL-21 treatment sustains metabolic quiescence and CD8^+^ T cell stemness, whereas IL-2 promotes glucose consumption and terminal differentiation. Accordingly, IL-21–treated CD8^+^ T cells better control tumor growth in adoptive cell therapy (ACT) (Hermans et al., 2020). Further, IL-2, in concert with TCR, enhances mTORC1 signaling, which is necessary for lipid synthesis from glucose in Treg cells (Zeng et al., 2013). Thus, Signal 3 cytokines play a role in coordinating nutrient uptake and metabolic rewiring with T cell fate.

### Amino acids

Amino acid uptake, synthesis, and sensing help orchestrate the generation of CD4^+^ T cell subsets (Fig. 2 B). Treatment of CD4^+^ T cells with halofuginone, a small molecule synthetic derivative of the natural product febrifugine, activates the amino acid starvation response and impairs Th17 cell differentiation, a process that is mimicked by depletion of amino acids (Sundrud et al., 2009). Arginine is a major precursor for the biosynthesis of polyamines, and polyamine synthesis is modulated by CD4^+^ T cell subset–polarizing cytokines (Signal 3), with the highest synthesis observed in Th1 and Th2 cells and lowest in Th17 cells. Further downstream, the synthesis of the amino acid hypusine from polyamines affects epigenetic regulation and CD4^+^ T cell subset differentiation, and disruption of the polyamine–hypusine axis broadly dysregulates the expression of cytokines and transcription factors by CD4^+^ T cell subsets (Puleston et al., 2021). Th17 cells are further divided into pathogenic and non-pathogenic (homeostatic) Th17 cells based on the ability to promote tissue inflammation, and these populations are marked by unique glycolytic and lipid metabolic pathways (Wang et al., 2015a; Wu et al., 2020a). There are alterations in amino acid metabolism among these subsets, with the polyamine pathway upregulated in pathogenic Th17 cells, whereas arginine synthesis and accumulation are higher in non-pathogenic Th17 cells (Puleston et al., 2021; Wagner et al., 2021). Consistent with these findings, disruption of the polyamine pathway promotes a Treg-like state in cells under Th17-skewing conditions and reduces autoimmunity (Wagner et al., 2021). Blocking extracellular polyamine uptake and intracellular de novo synthesis show coordinated effects in ameliorating EAE disease progression (Wu et al., 2020b), and the translational applications of targeting polyamine metabolism warrant further examination.

In addition to arginine, leucine and glutamine promote Th1 and Th17 cell differentiation (Nakaya et al., 2014; Sinclair et al., 2013). Glutamine and α-KG enhance the expression of IL-2–regulated Th1 effector genes by regulating DNA and histone methylation (Chisolm et al., 2017). In contrast to Th1 cells, Th17 cells rely on lower levels of glutamine for efficient differentiation, while elevated leucine levels promote Th17 and impair Th1 cell differentiation (Nakaya et al., 2014), suggesting differential dependencies on these amino acids. Further, in vitro glutamine deprivation impairs Th1 but promotes iTreg cell differentiation (via reducing α-KG) (Klysz et al., 2015), which may occur, in part, through the altered dependence of these subsets on mTORC1 signaling (Huang et al., 2020). In contrast, disruption of glutamine metabolism via pharmacological inhibition or genetic deletion of GLS impairs Th17 but promotes Th1 cell generation without impacting iTreg cell formation (Johnson et al., 2018). Further, methionine promotes the expansion of inflammatory Th17 cell–mediated EAE disease progression by epigenetically regulating histone H3K4 methylation at the promoter regions of key regulatory genes in Th17 cells (Roy et al., 2020). Thus, amino acids and associated metabolism play distinct and complex roles in directing subset specification of CD4^+^ T cells.

Branched-chain amino acids (BCAAs) (i.e., leucine, isoleucine, and valine) are essential for cell growth and function. TCR signaling induces the expression of the cytosolic branched-chain aminotransferase (BCATc), a BCAA catabolic enzyme, in CD4^+^ T cells, and this correlates with increased leucine metabolism by activated CD4^+^ T cells. BCATc deletion results in increased intracellular leucine levels and mTORC1 activation (Ananieva et al., 2014), providing a regulatory mechanism to alter mTORC1 activity in T cells. In addition, the uptake of BCAAs by SLC3A2 promotes the maintenance and proliferative state of Treg cells in vivo. Mechanistically, SLC3A2 deficiency or isoleucine-deficient conditions in vitro results in impaired mTORC1 signaling and cellular metabolism (Ikeda et al., 2017). In addition to amino acid uptake, intracellular amino acids derived via autophagy regulate T cell activation and function. For instance, TAX1BP1, a ubiquitin-binding protein, drives a specialized form of autophagy and promotes CD4^+^ T cell activation, likely by providing amino acids to promote mTORC1 signaling. Indeed, cysteine, but not leucine or methionine, supplementation in vitro rescues the proliferation defects of TAX1BP1-deficient CD4^+^ T cells (Whang et al., 2017). Altogether, these findings have illustrated multiple mechanisms by which T cells acquire or generate intracellular amino acids to support mTORC1 activity.

Serine, a non-essential amino acid, is crucial for effector CD4^+^ and CD8^+^ T cell responses. Serine is de novo synthesized or taken up from the extracellular environment (Pearce et al., 2013). Mechanistically, serine metabolism provides glycine and one-carbon units for purine nucleotide biosynthesis that is required for optimal T cell proliferation (Ma et al., 2017). Further, dietary restriction of serine results in limited CD8^+^ T cell responses to *Listeria monocytogenes* infection and failed control of bacterial growth (Ma et al., 2017), although proliferating CD8^+^ T cells in vivo predominantly rely upon de novo serine biosynthesis from glucose (Ma et al., 2019a). These effects are mediated, in part, by the capacity of serine to serve as an important nutrient input for one-carbon metabolism, the inhibition of which impairs T cell survival and antigen-driven T cell expansion (Ron-Harel et al., 2016). Additionally, MTHFD2, an enzyme in one-carbon metabolism, promotes de novo purine synthesis and effector T cell proliferation (Sugiura et al., 2022). Interestingly, under conditions of lactate-induced reductive stress, supplementation of serine augments T cell proliferation (Quinn et al., 2020), which may offer a therapeutic approach for restoring T cell–mediated immunity in immunosuppressive tissue microenvironments, such as the tumor microenvironment (TME) with high lactate levels (Apostolova and Pearce, 2022). Indeed, supplementation of formate, an intermediate in one-carbon metabolism downstream of serine, in combination with anti-PD-1 therapy, enhances CD8^+^ T cell accumulation in the TME and mediates better control of tumor growth (Rowe et al., 2023).

Amino acid uptake by activated T cells contributes to effector versus memory CD8^+^ T cell fate decisions. Genetic deletion of glutamine and arginine transporters, SLC38A2 and SLC7A1, respectively, enhances the proportion of memory precursor CD8^+^ T cells and the generation of memory T cells following LCMV infection (Huang et al., 2021). These effects are due to reduced mTORC1 signaling (Huang et al., 2021), consistent with enhanced memory formation upon in vivo rapamycin or metformin treatment (Araki et al., 2009; Pearce et al., 2009). Further, asymmetric cell division of activated T cells may “imprint” cells for effector versus memory cell fate. During asymmetric cell division, amino acid transporters, mTOR, and c-MYC preferentially segregate into the proximal daughter cell near the T cell–APC immunological synapse, while the distal daughter cells inherit fewer of these molecules (Chang et al., 2007; Pollizzi et al., 2016; Verbist et al., 2016). Further, proximal daughter cells have higher levels of the mTORC1–eIF2A complex that facilitates c-MYC synthesis (Liedmann et al., 2022). Higher expression of these proteins in the proximal daughter cell impairs long-term survival, while the distal daughter cell persists and better develops into memory T cells (Chang et al., 2007; Liedmann et al., 2022; Pollizzi et al., 2016; Verbist et al., 2016). Collectively, alterations in amino acid transporter expression during T cell division result in early changes in mTOR and c-MYC signaling and downstream metabolic programming (i.e., glycolysis versus mitochondrial metabolism) that regulate effector versus memory T cell fate decisions.

### Fatty acids

Fatty acids are classified into short-chain, medium-chain, and long-chain fatty acids (LCFAs) based on the length of the aliphatic chain (Fig. 2 C). Short-chain fatty acids (SCFAs) include propionate, acetate, and butyrate. Fermentation of dietary fiber by intestinal microbiota produces SCFAs, which impact the immune response in both the gastrointestinal tract and peripheral tissues. SCFAs generated by gut microbiota limit proinflammatory immune responses and help maintain intestinal homeostasis through GPR43 (also known as FFAR2) (Maslowski et al., 2009; Sun et al., 2018). In addition, dietary SCFAs promote CD8^+^ T cell antiviral responses in a GPR41-dependent manner, potentially by enhancing FAO in CD8^+^ T cells (Trompette et al., 2018). Further, butyrate promotes FOXO1 expression and formation of memory precursor CD8^+^ T cells after pathogen infection by enhancing FAO and mitochondrial metabolism (Bachem et al., 2019). Butryate also promotes IL-12 signaling in CD8^+^ T cells by increasing IL-12 receptor expression via the transcriptional regulator ID2, thus boosting effector function and enhancing antitumor activity (He et al., 2021). During systemic bacterial infection, serum acetate is increased and taken up by CD8^+^ T cells to promote acetylation of GAPDH and glycolysis-driven recall responses (Balmer et al., 2016). Conversely, acetate accumulation within inflamed tissues promotes glutaminolysis in CD8^+^ T cells, while suppressing TCR-mediated calcium flux and effector cell function, thereby enhancing their survival. The higher accumulation of acetate in inflamed tissues relative to serum suggests dose-dependent and microenvironmental effects on CD8^+^ T cell metabolic profiles and function (Balmer et al., 2020). Accordingly, in the context of nonalcoholic steatohepatitis (NASH), a systemic metabolic disease that causes liver damage, acetate promotes the auto-aggressive phenotype of CXCR6^+^ CD8^+^ T cells sensitized by IL-15 signaling and low FOXO1 expression (Dudek et al., 2021). A recent study has shown that dietary trans-vaccenic acid (TVA), a LCFA, disrupts SCFA signaling through GPR43, thereby promoting the cAMP–PKA–CREB axis that enhances CD8^+^ T cell function and antitumor immunity (Fan et al., 2023). Further, the LCFA linoleic acid promotes CD8^+^ T cell mitochondrial fitness by promoting ER–mitochondria contacts, leading to increased CD8^+^ T cell effector function (Nava Lauson et al., 2023). Thus, SCFAs and LCFAs have distinct effects on CD8^+^ T cell responses depending on the tissue and inflammatory contexts.

Fatty acids also contribute to cellular adaptation in environments that induce metabolic stress. Glucose-restricted CD8^+^ T cells catabolize acetate into acetyl-CoA through acyl-coenzyme A synthetase short-chain family member 2 (ACSS2) to promote chromatin accessibility and IFN-γ production in the TME (Qiu et al., 2019). Deleting ACSS2 in tumor cells results in a switch from acetate consumption to acetate production by tumor cells, thereby increasing acetate as a fuel source for tumor-infiltrating CD8^+^ T cells and enhancing T cell proliferation and effector function (Miller et al., 2023). Further, CD8^+^ T cells in tumors upregulate acetate metabolism upon glutamine blockade to generate acetyl-CoA that fuels the TCA cycle (Leone et al., 2019). Finally, increased potassium in the TME impairs the uptake of nutrients by T cells, consequently inducing their adaptation by upregulating autophagy and ACSS1-mediated acetyl-CoA production from acetate to promote mitochondrial metabolism and preserve T cell stemness and antitumor activity (Vodnala et al., 2019). These studies establish acetate as a key SCFA that supports T cell metabolic adaptation in nutrient-restricted microenvironments.

Beyond CD8^+^ T cells, SCFAs influence CD4^+^ T cell subsets by functioning as histone deacetylase (HDAC) inhibitors or binding to and signaling through GPCRs. Microbiota-derived butyrate and propionate, but not acetate, promote the differentiation of CD4^+^ T cells into peripherally induced Treg (pTreg) cells in vivo by epigenetically regulating *Foxp3* expression (Arpaia et al., 2013; Furusawa et al., 2013). Additionally, acetate and propionate increase pTreg numbers in the colon by activating GPR43 (Smith et al., 2013). Acetate, propionate, and butyrate promote IL-22 production by CD4^+^ T cells and innate lymphoid cells in the intestine via GPR41 signaling, and IL-22 production helps to maintain intestinal homeostasis (Yang et al., 2020). Further, treatment with SCFAs ameliorates EAE disease progression, potentially by affecting Treg cell function, while dietary LCFAs accelerate EAE by expanding pathogenic Th1 and Th17 cells (Haghikia et al., 2015). SCFAs also promote Th17, Th1, and IL-10^+^ CD4^+^ T cell differentiation by inhibiting HDAC-mediated suppression of mTOR activity (Park et al., 2015), as well as IL-10 production by Th1 cells by upregulating Blimp-1 expression (Sun et al., 2018). These findings highlight that SCFAs have discrete modes of action to influence CD4^+^ T cell differentiation.

Memory T cell subsets have distinct and tissue-specific usage of fatty acids. Memory CD8^+^ T cells take up fewer exogenous LCFAs compared with effector CD8^+^ T cells (O’Sullivan et al., 2014). Further, CD8^+^ T cells found in the circulation or secondary lymphoid tissues may depend on intracellular synthesis and lipolysis of fatty acids to fuel FAO for memory cell development and survival (Cui et al., 2015; O’Sullivan et al., 2014); however, genetic targeting of CPT1a, the rate-limiting enzyme for long-chain FAO, does not affect CD8^+^ memory T cell formation or recall responses in the spleen (Raud et al., 2018), suggesting alternative nutrients or carbon sources may fuel OXPHOS for memory T cells in secondary lymphoid tissues. Interestingly, Tem (which circulate and patrol peripheral tissues), central memory (Tcm, which reside in secondary lymphoid tissues), and tissue-resident memory (Trm, which reside long-term in tissues without recirculating) CD8^+^ T cells show altered dependence on different nutrients and metabolic pathways. In particular, Trm cells require exogenous free fatty acid uptake to promote oxidative metabolism and cell longevity (Frizzell et al., 2020; Lin et al., 2020; Pan et al., 2017). Trm cells have different expression profiles and requirements of fatty acid–binding proteins (FABPs) to maintain homeostasis (Frizzell et al., 2020). Specifically, skin CD8^+^ Trm cells rely on FABP4 and FABP5 (Pan et al., 2017), whereas liver Trm cells depend upon FABP1 (Frizzell et al., 2020). Further, memory CD8^+^ T cells in white adipose tissue (WAT) take up more LCFAs compared with memory T cells from the spleen or small intestine; however, during recall responses, these WAT-associated memory T cells show reduced lipid metabolism for functional responses (Han et al., 2017). Collectively, these studies support various requirements for fatty acids in memory T cell formation and maintenance within different tissue microenvironments.

### Cholesterol, bile acids, and other derivatives

As described above, cholesterol modulates T cell activation by interplaying with TCR signaling. Cholesterol-derived bile acids are produced in the liver and secreted into the duodenum. Bacteria in the intestine convert bile acids into secondary bile acids, including lithocholic acid (LCA). LCA-derived metabolites, such as 3-oxoLCA and isoalloLCA, have different roles in regulating Th17 or Treg cell differentiation (Fig. 2 D). 3-oxoLCA and isoLCA inhibit Th17 cell differentiation by binding to and inhibiting RORγt activity, a key transcription factor for Th17 cells (Hang et al., 2019; Paik et al., 2022). In contrast, bile acid metabolites, including 3-oxoLCA, promote RORγt-expressing pTreg cell accumulation in the colon through the vitamin D receptor (VDR) (Song et al., 2020), whereas the induction of pTreg cells by isoalloLCA is partly dependent on the nuclear hormone receptor NR4A1 (Li et al., 2021). Further, isoalloLCA promotes FOXP3 expression for pTreg cell generation in a conserved non-coding sequence 3–dependent manner, and dietary supplementation of isoalloLCA enhances intestinal Treg cell accumulation (Hang et al., 2019). In addition, the bile acid metabolite 3β-hydroxydeoxycholic acid (isoDCA) also induces FOXP3 expression, albeit indirectly by impairing DC function (Campbell et al., 2020). To minimize the toxicity of bile acids, effector CD4^+^ T cells in the small intestine lamina propria express the constitutive androstane receptor (CAR), a nuclear xenobiotic receptor. CAR promotes the expression of detoxifying enzymes and transporters in CD4^+^ T cells, as well as the anti-inflammatory cytokine IL-10, together helping to resolve inflammation (Chen et al., 2021). Thus, targeting bile acids or downstream signaling modulates T cell responses and intestinal inflammation. Other cholesterol derivatives (e.g., oxysterols [Li et al., 2016]) or membrane lipids (e.g., phosphatidylethanolamine [Fu et al., 2021]) are important for T cell differentiation as reviewed elsewhere (Lim et al., 2022).

## Nutrient deprivation and suppression of T cell function

Similar to coinhibitory receptors (Signal 2) and immunosuppressive cytokines (Signal 3) that inhibit T cell activation and effector functions (Giles et al., 2023), inhibitory nutrients (Signal 4) or nutrient deprivation exerts immunosuppressive effects on T cell responses. In tissue microenvironments, reduced nutrient availability due to intercellular competition or nutrient-mediated induction of immunosuppressive cell subsets (e.g., Treg cells) impacts T cell fate and function. Further, aberrant uptake and accumulation of certain nutrients, such as lactate, oxidized lipids, and cholesterol, induce CD8^+^ T cell dysfunction in the TME. Here, we summarize how nutrient deprivation or certain inhibitory nutrients limit T cell function.

### Glucose and amino acid deprivation and dysregulation

**Figure 3.**
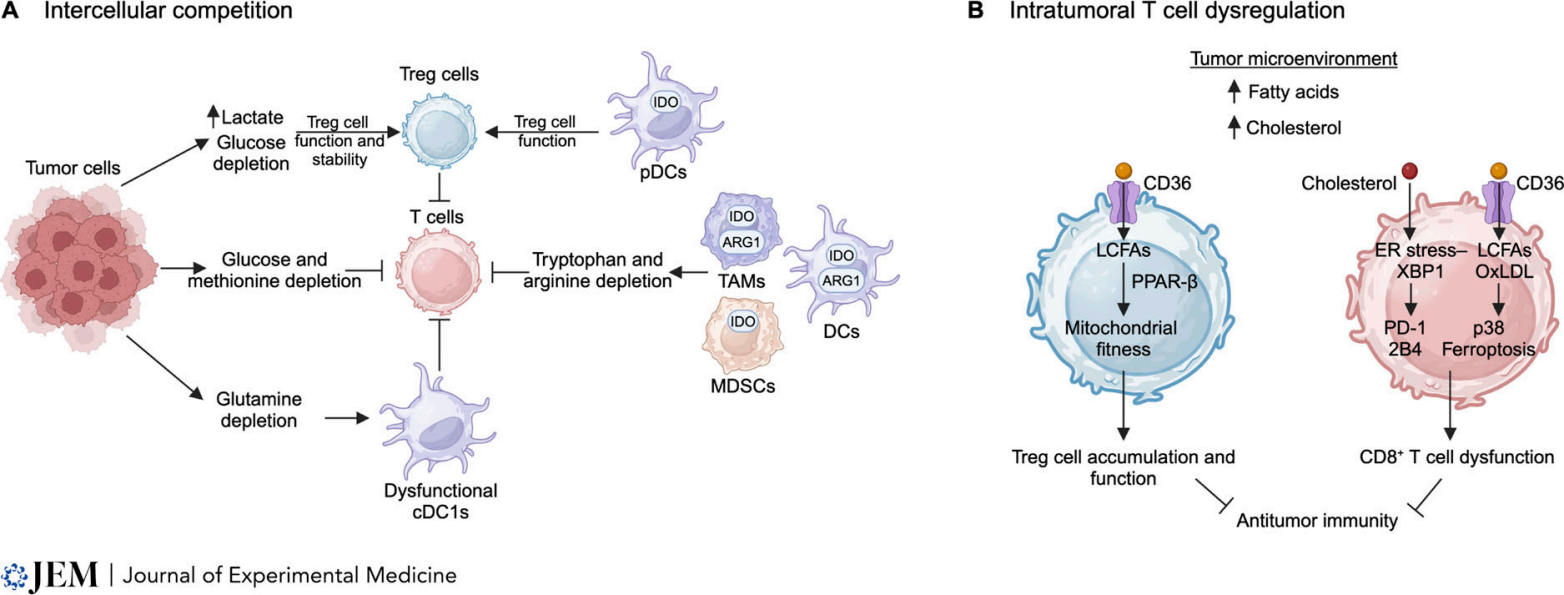
**Nutrient deprivation and lipid accumulation limit T cell function. (A)** Intercellular competition for nutrients can limit T cell function. Tumor cells compete with immune cells for glucose, glutamine, and methionine in the TME, leading to nutrient deprivation that directly inhibits T cells, or indirectly inhibits T cells by negatively affecting the functionality of cDC1s. In contrast, metabolic adaptation of Treg cells allow the cells to maintain their suppressive capacity in conditions of low glucose and high lactate in the TME. IDO-expressing pDCs help maintain Treg suppressive function. Further, IDO and ARG1-expressing DCs and TAMs, and IDO-expressing MDSCs, catabolize tryptophan and arginine, leading to localized depletion. Consequently, T cell function is impaired. **(B)** Lipids, including fatty acids and cholesterol, accumulate in the TME. CD8^+^ T cells in the TME increase the uptake of oxidized low-density lipoprotein (OxLDL) by CD36, leading to greater lipid peroxidation, p38 kinase activation and ferroptosis. Further, increased intracellular cholesterol in CD8^+^ T cells promotes ER stress–XBP1 signaling and coinhibitory receptor expression, including PD-1 and 2B4. Together, increased cholesterol and fatty acids induce CD8^+^ T cell dysfunction in the TME. However, increased CD36 expression on intratumoral Treg cells correlates with increased lipid uptake and mitochondrial fitness and persistence via PPAR-β signaling. Treg cell accumulation in the TME may further impair T cell function and antitumor immunity.

Competition for glucose between tumor cells and T cells in the TME plays an important role in tumor growth and immune evasion (Fig. 3 A). Similar to activated T cells, tumor cells increase glucose uptake and aerobic glycolysis, leading to reduced levels of glucose in the TME that impair T cell effector function (Chang et al., 2015; Ho et al., 2015), with multiple possible mechanisms involved. First, in the absence of aerobic glycolysis, T cells produce less IFN-γ due to GAPDH-mediated inhibition of *Ifng* mRNA translation (Chang et al., 2013) or defective epigenetic regulation via histone acetylation (Peng et al., 2016). Second, under glucose restriction, the glycolytic metabolite phosphoenolpyruvate (PEP) is reduced, leading to reduced cytosolic calcium levels that are required to sustain TCR-mediated calcium–NFAT signaling and T cell responses in the TME (Ho et al., 2015). Third, intratumoral CD8^+^ T cells downregulate ENOLASE 1 activity, which promotes cellular dysfunction (Gemta et al., 2019). Further, CD8^+^ T cell glycolysis and effector function are disrupted by the oncometabolite D-2-hydroxyglutarate secreted by tumors with isocitrate dehydrogenase mutations, leading to impaired antitumor effects (Bunse et al., 2018; Notarangelo et al., 2022). Importantly, immune checkpoint blockade (ICB) treatments help restore T cell function in the TME, in part, by rectifying defects in intracellular glucose levels and glucose metabolism (Chang et al., 2015). In contrast to CD8^+^ T cells, low glucose conditions or uptake promotes Treg cell function and stability in vitro and in vivo (Gerriets et al., 2016; Watson et al., 2021; Zappasodi et al., 2021). Thus, modulating glucose availability or glycolysis in T cells may boost antitumor immunity by promoting intratumoral CD8^+^ T cell and inhibiting Treg cell functions.

Glycolytic tumors secrete the metabolite lactate, which influences the function of T cells within the tumor microenvironment. High lactate levels in the TME rewire pyruvate metabolism in CD8^+^ T cells, leading to reduced succinate secretion by CD8^+^ T cells and autocrine signaling through the succinate receptor (SUCNR1). Reduced succinate–SUCNR1 signaling impairs CD8^+^ T cell cytotoxic function (Elia et al., 2022). Further, lactate uptake by CD8^+^ T cells causes intracellular acidification and impairs NFAT signaling (Brand et al., 2016). Treating CD8^+^ T cells with sodium bicarbonate reduces intracellular acidification caused by lactate, thus restoring metabolic fitness and enhancing graft-versus-leukemia responses in mice and humans (Kumagai et al., 2022; Watson et al., 2021). However, administration of sodium lactate can promote antitumor immunity by enhancing CD8^+^ T cell stemness, and this effect may be due to the use of sodium lactate that maintains a neutral pH as compared with the acidic pH induced by lactic acid (Feng et al., 2022). Unlike CD8^+^ T cells, Treg cells can acquire and metabolize lactate, espeically in the TME (Angelin et al., 2017; Watson et al., 2021). The lactate transporter MCT-1 in Treg cells is important for intratumoral Treg cell function (Kumagai et al., 2022). Reducing lactate abundance or uptake may therefore potentiate CD8^+^ T cells while suppressing Treg cell function in glycolytic tumors, suggesting the potential for therapeutic intervention.

Consumption of amino acids in tissue microenvironments suppresses local T cell responses. In addition to consuming glucose, tumor cells take up amino acids, including glutamine and methionine, contributing to the depletion of these amino acids in the TME. Such depletion directly impairs T cell function (Bian et al., 2020; Hung et al., 2021; Pandit et al., 2023) or acts indirectly to impair T cell reactivation in the TME by affecting cDC1 function (Guo et al., 2023). Using positron emission tomography, a recent study showed that tumor cells take up more glutamine and less glucose compared with immune cells in the TME, whereas myeloid cells have the greatest capacity to use glucose (Reinfeld et al., 2021). Further, immunosuppressive myeloid populations consume and deplete extracellular arginine, leading to impaired T cell proliferation (Fletcher et al., 2015; Mondanelli et al., 2017; Norian et al., 2009; Rodriguez et al., 2003, 2004; Zea et al., 2005). Such cell cycle arrest is reversible with exogenous arginine treatment (Van de Velde et al., 2017), and arginine supports activated T cell survival and antitumor function in a model of ACT (Geiger et al., 2016), thus providing a therapeutic approach to overcome cellular dysfunction. The ability of T cells to sense low levels of amino acid availability can serve as a mechanism to adapt to nutrient depletion in the TME, for example, by activating GCN2 in murine models of malignant glioma (Rashidi et al., 2020). Altogether, targeting amino acid consumption by tumor and myeloid cells can mitigate T cell dysfunction in tumors.

IDO is a metabolic enzyme that catabolizes the amino acid tryptophan and is expressed by various cell types, including intratumoral myeloid cells. T cells require tryptophan for proliferation and activation, and localized depletion of tryptophan by IDO inhibits T cell responses (Kelly and Pearce, 2020; Munn et al., 2005). Further, catabolized tryptophan generates the metabolite 3-hydroxyanthranillic acid (3-HAA), and 3-HAA promotes iTreg cell differentiation by increasing TGF-β production by DCs (Yan et al., 2010). In addition, kynurenine, a product of IDO-mediated catabolism of tryptophan, is transported into T cells by SLC7A5 and activates aryl hydrocarbon receptor signaling (Sinclair et al., 2018), which enhances iTreg cell differentiation (Mezrich et al., 2010). Blocking IDO promotes IL-6 production by plasmacytoid DCs (pDC) and induces IL-17 production by Treg cells in vivo (Baban et al., 2009; Sharma et al., 2009). IDO-deficient mice have accelerated EAE disease progression along with increased Th17 cells and reduced Treg cells, and treatment with 3-HAA rectifies disease progression and the altered Th subset skewing (Yan et al., 2010). Further, IDO-expressing pDCs in the tumor-draining lymph nodes induce T cell anergy (Friberg et al., 2002; Munn et al., 2004). Additionally, IDO-expressing tumor-associated macrophages (TAMs) and myeloid-derived suppressor cells (MDSCs) deplete tryptophan and consequently enhance CD8^+^ T cell dysfunction in the TME (Kao et al., 2022). Collectively, limiting tryptophan in the microenvironment has an overall immunosuppressive effect, favoring Treg cell generation or expansion at the expense of effector T cell responses.

### Lipids in tissue microenvironments

The TME is enriched with lipids that have immunosuppressive effects on T cell function, thereby promoting tumor immune evasion (Fig. 3 B). CD36 is a scavenger receptor responsible for the uptake of LCFAs and oxidized low-protein lipoproteins (LDL), and intratumoral T cells upregulate the expression of CD36. Increased CD36 expression on intratumoral Treg cells correlates with increased lipid uptake and metabolism, enhancing Treg cell accumulation and suppressive function. Mechanistically, CD36 promotes mitochondrial fitness and persistence of intratumoral Treg cells via PPAR-β (Wang et al., 2020a). Unlike Treg cells, CD36 expression on intratumoral CD8^+^ T cells contributes to their dysfunction (Ma et al., 2021; Xu et al., 2021). CD8^+^ T cells in the TME increase the uptake of oxidized LDL by CD36, leading to greater lipid peroxidation and p38 kinase activation (Xu et al., 2021). Further, intratumoral fatty acids induce ferroptosis in CD8^+^ T cells in a CD36-dependent manner (Ma et al., 2021). Targeting CD36 or ferroptosis promotes CD8^+^ T cell function against tumors (Ma et al., 2021; Xu et al., 2021). In pancreatic ductal adenocarcinoma, intratumoral CD8^+^ T cells accumulate intracellular LCFAs that contribute to impaired mitochondrial and cellular function (Manzo et al., 2020). Further, obesity is a cancer risk factor, and mice fed with high-fat diets (HFDs) have accelerated growth associated with enhanced T cell dysfunction (Dyck et al., 2022; Ringel et al., 2020; Wang et al., 2019; Zhang et al., 2020a). In mice fed with HFDs, deleting STAT3 enhances intratumoral CD8^+^ T cell function by reducing FAO and promoting glycolysis in a model of breast cancer (Zhang et al., 2020a). In contrast, subcutaneously transplanted tumor cells in mice fed with HFDs outcompete CD8^+^ T cells for lipids within the TME, resulting in reduced fatty acid uptake by intratumoral CD8^+^ T cells and impaired CD8^+^ T cell function (Ringel et al., 2020), suggesting context-dependent effects of extrinsic lipids on intratumoral CD8 T cell function under HFDs. Together, these findings highlight that targeting lipid availability, uptake, or metabolism may overcome T cell dysfunction in certain tumor contexts by acting on both CD8^+^ T cells and Treg cells in the TME.

Exogenous cholesterol impacts T cell homeostasis in different disease settings. For instance, hypercholesterolemia in humanized mice increases Tem cells, whereas Treg cells are reduced (Proto et al., 2018). In the context of tumors, cholesterol in the TME promotes T cell exhaustion, a cell state characterized by high expression of inhibitory receptors and impaired function, by activating the ER stress–XBP1 pathway (Ma et al., 2019b). Deletion of XBP1 improves T cell function in the TME (Song et al., 2018). Further, cholesterol inhibits IL-9 expression in IL-9–secreting CD8^+^ T cells (called Tc9 cells), a cell subset that has strong antitumor effects in ACT models (Ma et al., 2018b). Interestingly, recent findings highlight that oxysterols in the TME reduce intracellular cholesterol levels in CD8^+^ T cells, likely by impairing SREPB2 and promoting LXR activity, and deletion of LXRβ in chimeric antigen receptor T (CAR-T) cells promotes antitumor immunity (Yan et al., 2023). Intratumoral CD8^+^ T cells downregulate expression of LDL receptor (LDLR), which takes up LDL that contains cholesterol. LDLR deficiency impairs CD8^+^ T cell function, as LDLR potentiates TCR recycling and signaling at the plasma membrane (Yuan et al., 2021). Dietary cholesterol also influences immune cell function. Indeed, high cholesterol in the diet, a feature of the Western diet, impairs the formation of CD8^+^ Trm cells in the small intestine by suppressing SREBP2 activity and the downstream generation of non-steroidal metabolites from the mevalonate-cholesterol synthesis pathway. Of note, this is a common mechanism for both small intestinal Trm cells and intratumoral Trm-like CD8^+^ T cells (Reina-Campos et al., 2023). Together, these studies illustrate diverse effects of exogenous cholesterol on T cell function that may be targeted for immunotherapy.

## Modulating nutrients for disease therapy

Nutritional interventions have shown promising effects on patient outcomes for different diseases, including infection, autoimmunity, and cancer (Collins and Belkaid, 2022; Kao et al., 2022). Experimental and clinical evidence that targeting nutrients along with those boosting Signals 1–3 (e.g., anti-PD-1 or -PD-L1 antibody blockade to increase Signal 2) is a promising combinatorial approach for treatment. Here, we summarize the impact of dietary interventions or direct nutrient targeting on disease outcomes.

### Dietary interventions

**Figure 4.**
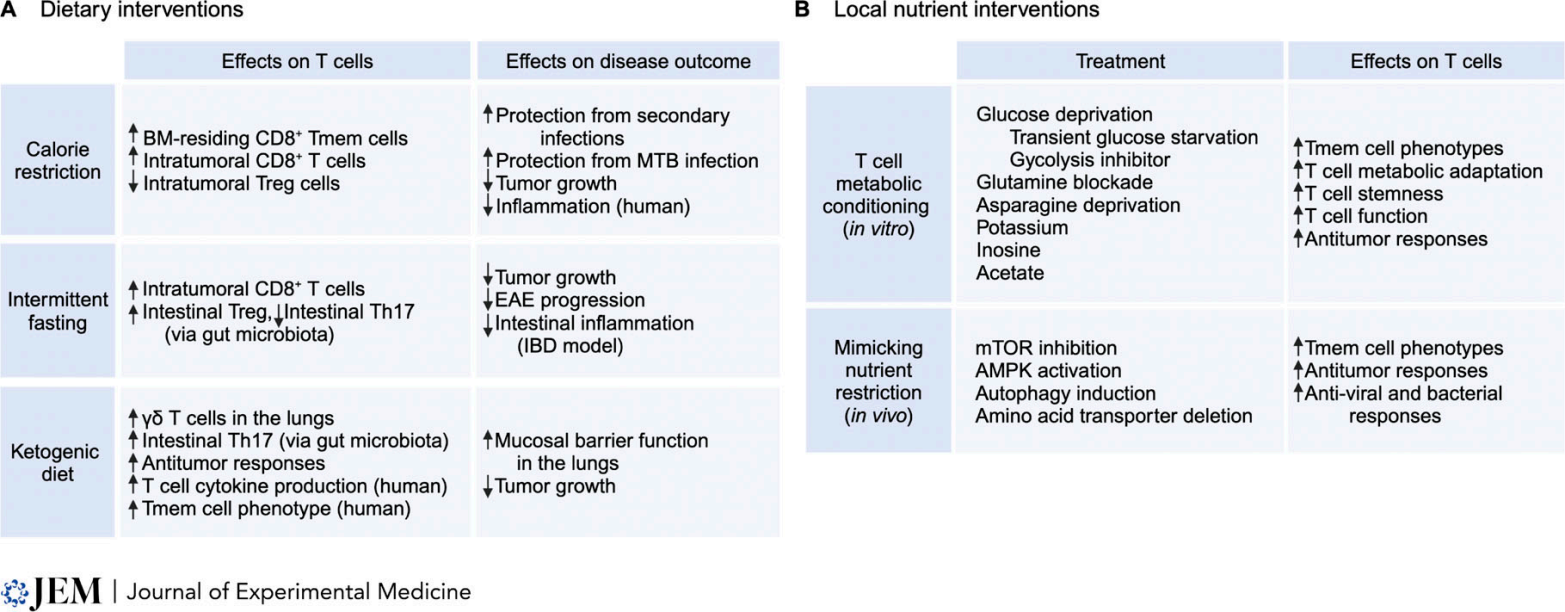
**Nutritional intervention for disease therapy. (A)** The effects of calorie restriction, intermittent fasting, or the ketogenic diet on T cell immunity and disease outcomes in mice and humans. **(B)** The effects of in vitro metabolic conditioning, including glucose deprivation, glutamine blockade, asparagine deprivation, or supplementation with potassium, inosine, and acetate, on CD8^+^ T cell fate and function in vivo (top row). The effects of in vivo treatments that mimic calorie restriction, including mTOR inhibition, AMPK activation, induction of autophagy, and deletion of amino acid transporters, on T cell fate and function. Bone marrow (BM), *Mycobacterium tuberculosis* (MTB), experimental autoimmune encephalomyelitis (EAE), inflammatory bowel disease (IBD).

Dietary interventions include calorie restriction, nutrient-specific restriction, and fasting (Collins and Belkaid, 2022) (Fig. 4 A). Calorie restriction promotes the accumulation of functional memory T cells in the bone marrow (BM) during *Yersinia pseudotuberculosis* infection, but reduces memory T cell accumulation in secondary lymphoid tissues and WAT, suggesting a unique role for the BM microenvironment in maintaining the memory T cell pool during nutritional stress (Collins et al., 2019). Additionally, calorie restriction protects mice from pulmonary infection of *Mycobacterium tuberculosis* (MTB) (Palma et al., 2021), with calorie restriction lowering mTOR signaling in T cells (Collins et al., 2019; Palma et al., 2021), in line with the observations that reducing mTOR signaling favors memory T cell development and persistence (Araki et al., 2009). Interestingly, microbiota contributes to enhanced memory CD8^+^ T cell function in mice fed with calorie-restricted diets (Han et al., 2023). A mechanistic understanding of how microbiome and calorie restriction cooperate to promote memory T cell function is of great interest for future studies.

In the context of tumors, feeding tumor-bearing mice diets that mimic intermittent fasting alone or in combination with chemotherapy increases the number of intratumoral CD8^+^ T cells and reduces tumor growth (Di Biase et al., 2016) and metastasis in a mouse model of breast cancer (Pomatto-Watson et al., 2021). Further, calorie restriction in combination with radiation or chemotherapy lowers intratumoral Treg cells and expands CD8^+^ T cells in tumor-bearing mice (Manukian et al., 2021; Pietrocola et al., 2016), and human patients with breast cancer undergoing a similar treatment have reduced immunosuppressive cytokine levels in the serum (Manukian et al., 2021). Additional human studies have reported that dietary restriction inhibits inflammation, while still preserving or even enhancing T cell function (Ahmed et al., 2009; Meydani et al., 2016). It is important to note that consumption of artificial sweeteners when attempting to lower caloric intake may negatively impact adaptive immunity, as intake of high sucralose levels by mice impairs TCR signaling and subsequent T cell proliferation and differentiation (Zani et al., 2023). One potential downside of dietary restriction is the increase in levels of glucocorticoids (Collins et al., 2019), and long-term exposure to glucocorticoids has pleiotropic immunosuppressive effects on adaptive immunity (Cain and Cidlowski, 2017). Thus, while moderate dietary restrictions (that do not induce malnutrition) may benefit the immune response to infection and cancer, more targeted dietary approaches are warranted for long-term interventions.

Nutrient restriction helps control aberrant inflammation. Intermittent fasting ameliorates disease progression and pathology of EAE. Mechanistically, intermittent fasting affects the intestinal microbiota diversity, which in turn promotes Treg cell and inhibits Th17 cell accumulation in vivo. Importantly, fecal transfer from mice with intermittent fasting is protective in EAE (Cignarella et al., 2018). A diet that mimics fasting also mitigates intestinal inflammation in a mouse model of inflammatory bowel disease, associated with altered intestinal microbiota and reduced CD4^+^ and CD8^+^ T cell accumulation in the intestine (Rangan et al., 2019). How the homeostasis of other tissues and the associated immune cell populations are affected by diet and nutrient restriction is an exciting area of future research.

The ketogenic diet, characterized by a very low abundance of carbohydrates and accumulation of ketone bodies, also influences T cell responses. For instance, γδ T cells are expanded in the lungs of mice fed with ketogenic diets, a phenotype that occurs during influenza A virus or coronavirus infection in aged mice (Goldberg et al., 2019; Ryu et al., 2021). These γδ T cells promote mucosal barrier integrity in the lungs that helps protect the mice from infection (Goldberg et al., 2019). Further, ketogenic diets decrease intestinal Th17 cells by altering the intestinal microbiota (Ang et al., 2020). Elevated ketone bodies generated in patients on the ketogenic diet also enhance T cell cytokine production and skew cells toward a memory T cell phenotype, associated with reduced glycolysis and elevated mitochondrial OXPHOS (Hirschberger et al., 2021). Production of ketone bodies such as β-hydroxybutyrate (BHB) is increased during infections. BHB promotes mitochondrial metabolism and CD4^+^ T cell effector function, and a reduction in BHB is observed in individuals with SARS-CoV-2-induced acute respiratory distress syndrome (Karagiannis et al., 2022). BHB also increases CD8^+^ T cell effector responses by generating acetyl-CoA to fuel the TCA cycle and promote histone acetylation of effector genes (Luda et al., 2023). Further, BHB promotes memory CD8^+^ T cell formation by enhancing the expression of *Foxo1* and *Ppargc1a* via epigenetic modifications. FOXO1 and PGC-1α cooperate to promote the expression of Pck1 (Zhang et al., 2020b). Pck1 is an enzyme that promotes gluconeogenesis to fuel the pentose phosphate pathway and generate NADPH, which protects against the accumulation of intracellular reactive oxygen species (ROS) (Ma et al., 2018a). The ketogenic diet also enhances antitumor immunity in combination with ICB (Dai et al., 2021; Ferrere et al., 2021). Together, these studies suggest a beneficial role of the ketogenic diet and ketone bodies on various disease outcomes.

Diets modified for specific nutrients or metabolites influence T cell responses. For instance, methionine-restricted diets reduce EAE disease severity through epigenetic regulation of inflammatory Th17 cells (Roy et al., 2020). Feeding mice with diets deficient in asparagine or serine and glycine limits early effector T cell activation and expansion during infection (Ma et al., 2017; Wu et al., 2021). Conversely, supplementing diets with arginine enhances activated T cell survival (Geiger et al., 2016). Further, supplementation of formate in drinking water promotes the efficacy of anti-PD-1 treatment to control tumor growth (Rowe et al., 2023). Supplementing diet with the LCFA TVA enhances CD8^+^ T cell function and antitumor immunity (Fan et al., 2023). Additionally, salt and sugar in the diet modulate the gut microbiome that influences Th17 formation. Specifically, diets high in salt potentiate Th17 cell differentiation and consequently accelerate EAE disease progression (Kleinewietfeld et al., 2013; Wu et al., 2013), associated with changes in the gut microbiome (Wilck et al., 2017). Dietary sugar displaces bacteria in the intestine that induce homeostatic Th17 cells, leading to intestinal inflammation and metabolic syndrome (Kawano et al., 2022). Together, these studies support the therapeutic approach of depleting or supplementing individual nutrients in the diet to modulate T cell immunity.

### Targeting nutrients and nutrient signaling

Harnessing the mechanistic effects of nutrient restriction on T cell physiology has promising therapeutic potential (Fig. 4 B). In vitro metabolic conditioning of T cells has positive effects on T cell persistence and functional capacities. For instance, activated CD8^+^ T cells cultured in the presence of 2-DG display increased mitochondrial metabolism that favors memory T cell formation and function. Adoptive transfer of these CD8^+^ T cells enhances antitumor immunity (Sukumar et al., 2013). Consistent with these findings, activated T cells transiently cultured with low glucose undergo metabolic adaptation that confers enhanced functional responses upon glucose re-exposure, thus allowing these cells to better control tumor growth after adoptive transfer (Klein Geltink et al., 2020). Further, the blockade of glutamine metabolism with the pro-drug JHU083 or 6-diazo-5-oxo-L-norleucine conditions CD8^+^ TILs for an activated, memory cell phenotype and results in enhanced antitumor responses (Leone et al., 2019). Supplementation of metabolites that promote T cell function under glucose and glutamine-restricted conditions, including inosine and acetate (Klysz et al., 2024; Leone et al., 2019; Mager et al., 2020; Qiu et al., 2019; Wang et al., 2020b), provides additional approaches to condition T cells for enhanced efficacy of ACT. Further, activated CD8^+^ T cells expanded under asparagine-restricted conditions ex vivo better control tumor growth in an ACT model by enhancing the NRF2-dependent stress response; this response facilitates nucleotide biosynthesis and cell proliferation (Gnanaprakasam et al., 2023). Finally, increasing arginine levels directly in the TME via engineered bacteria shows synergistic effects with PD-L1 antibody blockade (Canale et al., 2021), providing another combinatorial approach to improve antitumor immunity.

An alternative approach to nutrient interventions is targeting intracellular nutrient sensors and nutrient-responsive signaling pathways to mimic nutrient restriction. For instance, inhibiting mTORC1 signaling, by genetic approaches or pharmacological inhibitors, boosts memory T cell formation in the context of pathogen infection (Araki et al., 2009). Further, as aforementioned, reducing the availability of intracellular amino acids or targeting their transporters dampens mTORC1 signaling and enhances memory T cell development (Huang et al., 2021; Pollizzi et al., 2016; Verbist et al., 2016). Consistent with these findings, mTOR inhibitors enhance immune responses to vaccines in humans (Mannick et al., 2018). Further, autophagy promotes memory T cell formation (Puleston et al., 2014; Xu et al., 2014). High potassium concentration in the TME can promote a nutrient-restricted state in T cells that induces autophagy and enhances T cell stemness and persistence in the TME (Vodnala et al., 2019). However, promoting efflux of potassium in T cells by overexpressing the potassium channel enhances antitumor effector function of intratumoral T cells by restoring Akt–mTOR signaling (Eil et al., 2016), highlighting multifactorial roles for potassium in regulating the antitumor properties of CD8^+^ T cells. Recent studies have shown that myeloid cells, including TAMs and DCs, in the TME must regulate TFEB and TFE3 activity to overcome amino acid restriction and modulate antitumor immunity (Guo et al., 2023; Zhang et al., 2023). Interestingly, hyperactivation of TFEB in FLCN-deficient or glutamine-deprived DCs results in impaired priming of T cells (Guo et al., 2023), whereas TAMs require increased TFEB and TFE3 activity to impair tumor growth (Zhang et al., 2023). Whether targeting TFEB and TFE3 in T cells affects memory T cell formation or antitumor immunity remains to be explored. Collectively, these findings demonstrate that understanding the mechanisms by which cells adapt to nutrients and nutrient restriction may lead to the development of novel immunotherapies.

## Conclusions and future perspectives

Nutrients have emerged as Signal 4 that serve as a key driver of T cell activation, differentiation, and function. Investigations exploring the interplay of nutrients with Signals 1–3 (i.e., TCR, coreceptor, and cytokine receptor-mediated signaling) have uncovered the dependency of T cells on nutrient uptake, sensing, and signaling for cellular quiescence exit and entry into an activation state. Further, nutrients, in coordination with Signals 1–3, serve both proinflammatory and immunosuppressive roles that direct T cell fate and function and impact disease outcomes. How the three-tiered process of intracellular nutrient transport, sensing, and signaling is regulated in discrete tissue microenvironments and how these signaling events interplay with T cell activation, differentiation, and function is an exciting and active area of research in adaptive immunity. Moreover, an increased mechanistic understanding of how nutrients serve as Signal 4 in tissue and disease-specific contexts promises to uncover actionable targets for therapy.

Future studies are needed to provide functional and mechanistic insights as to how nutrients regulate T cell immunity in different tissue types and tissue microenvironmental niches. The application of new technologies and multiomics approaches will help expedite such investigations and our understanding of spatiotemporal regulation of adaptive immunity mediated by nutrients, especially with single-cell and spatial resolution. Indeed, both single-cell mass cytometry and flow cytometry–based approaches have uncovered key insights into the metabolic state of immune cells (Argüello et al., 2020; Hartmann et al., 2021; Levine et al., 2021). There are also computational and bioinformatic tools, including Compass and MetaFlux, that infer changes in metabolic activities based on gene expression in single-cell transcriptomic datasets that can be applied to identify metabolic programs underlying T cell responses (Huang et al., 2023; Wagner et al., 2021). Finally, spatial transcriptomic, proteomic, and metabolomic analysis (Mogilenko et al., 2023) tools are compelling approaches to understand how nutrients and metabolic programs orchestrate adaptive immunity in discrete locations within a tissue.

With the emergence of new functional screening platforms (Shi et al., 2023), we have an unprecedented ability to establish the causality of how Signal 4 orchestrates T cell immunity and interplays with traditional immunological signals in discrete tissue and disease contexts. For instance, the strategy of combining bulk CRISPR functional screening with metabolomics and transcriptomics uncovered a unique contribution of non-steroidal products from the mevalonate pathway to Trm cell formation (Reina-Campos et al., 2023). Further, our in vivo single-cell CRISPR screening study revealed metabolism-associated modalities that can be targeted to promote antitumor immunity and ICB efficacy by promoting quiescence exit and enriching the metabolically-active, proliferative state of intratumoral CD8^+^ T cells (Zhou et al., 2023). The extent to which nutrients are involved in shaping these intratumoral CD8^+^ T cell differentiation states or improving the responses to ICB will be important to explore. Harnessing functional genomics approaches to interrogate Signal 4, especially at the single-cell level and at high dimension, provides an excellent opportunity to discover new regulatory circuits in T cell immunity.

Nutritional intervention is emerging as a promising strategy to improve patient outcomes when used in combination with immunotherapy or other therapeutics. Nutrients can be targeted through dietary changes or local administration, or modulating the expression or activity of nutrient transporters, sensors, and signaling mediators, or the biosynthetic and catabolic processes. Also, metabolic conditioning of T cells ex vivo through nutrient supplementation or deprivation has potential as a therapeutic approach to enhance ACT, and the durability of such transient treatments on T cell function, and the underlying basis, will be interesting to explore. Further, while dietary nutrients have a pronounced impact on human physiology, we are only beginning to understand how nutritional restriction or dietary intervention affects the immune system. Given the detrimental effects of prolonged calorie restriction on overall host health (Collins and Belkaid, 2022), future investigations to establish targeted approaches that mimic the immunological benefits of calorie restriction are warranted. Altogether, these lines of future studies manifest the opportunity to advance and optimize therapies for infection, cancer, and autoimmunity, as well as provide strategies for vaccination.
